# Self – Regard of Individuals with Autism – How People from the Autism Spectrum Perceive Autism. A Netnographic Research

**DOI:** 10.1007/s12124-021-09601-3

**Published:** 2021-02-26

**Authors:** Jacek Jarosław Błeszyński

**Affiliations:** Faculty of Philosophy and Social Sciences, Nicolaus Copernicus University, Torun, Poland

**Keywords:** Autism spectrum disorders, Self-regard, Netnographic research, Inclusion

## Abstract

This research on adults with a diagnosis of autism is a multidirectional and multistage analysis. It is aimed at reviewing the current knowledge about the disorder as well as taking optimal actions towards social inclusion for people with ASD (autistic spectrum disorder). The research and analysis was facilitated by selecting subjects who have sufficient communication and social skills to be able to participate in the study. The new DSM 5, which now has descriptors of CTS (Conflict Tactics Scale) degrees, was useful for this purpose. Data was gathered by means of personalized forms and methods, with in-depth netnographic (rather than face-to-face) interviews conducted. As no similar research using the netnographic method could be found, a pilot study was initiated, which was then gradually expanded to include a larger number of respondents. This research made it possible to record the views of people with ASD regarding autism and their functioning in society.

## Introduction

It is important to change the perspective of the approach to cognition itself as a process of obtaining information, to realise the importance of using qualitative research in the search for subjective and terminated concepts of autism and autism spectrum disorders. The aim of the research was to analyse the reflection of ASD individuals on their condition. There had been no such focus on this prior to the author’s study. The research investigated reflection on autism, not by the author but by the subjects of the study.^1^ Therefore, reference should be made to the philosophy of cognition which is aimed at knowing the other person.

### Knowing the Other Person – from Hursserl to Węgrzecki’s View

Cognition, starting from the assumptions presented by Edmund Husserl, is the search for the possibility of crossing the boundaries of the psychophysical reflection of the view of the philosophy. E. Husserl noted that the ultimate phenomenological subject, which is not subject to any exclusion and is itself the subject of all eidetic-phenomenological research, is the pure self (Husserl 1974). It is only the cognition of the other person that can be based on the empathy for the other person (Einfühlung) as an indirect presence (Husserl 1974). As pointed out by E. Husserl, others can be experienced, but only by feeling their own content can they experience the source perception. The experiences of individuals are available to others only indirectly – through their experience (Husserl 1974). This approach is burdened with a subjectivity. It has been changed by Edith Stein, who noted that, as a unit, individuals are in the human community. Thus, empathy is a kind of experience in a specific space and time with material processes and mental experiences (Stein 1917). It is this perspective that broadens the field of experience and becomes a deepened element of understanding. Humans experience others not by the feeling of unity, but by feeling empathy. As a consequence, it becomes possible to feel unity and enrich one’s own experience (Stein 1917). On this basis, the awareness of physical (e.g. part of the body) and psychic (own and other sensations and thoughts) domain is created. “Expressive phenomena” is a feeling that allows one to penetrate the acts of another person, their specific sphere of being, which is personal spirituality.

Max Scheler created the theory of cognition, which is now the basis of approach to cognition in the humanities. The man, as a social being, perceives another man belonging to a specific sphere being defined as “the world of You (*Duwelt*)”. It is the interactionism that occurs within the community that shapes the way people interact with each other. As noted by Węgrzecki, living in community, an individual presents oneself through direct observation and co-experience. In contrast, for society, the separation of the “survival” and “bodily word movement” are a variable in other people (Węgrzecki 1972).

People build their experience of the world based on psychological experiences (from internal self-observation or someone else’s views). This process includes the self and the experience of another human being, just as self and experiencing in general includes not just the immediate present (Scheler 1972). Getting to know the other person does not happen properly if this process is not spontaneous or formed at an emotional level (Scheler 1972). The ability to love involves eliciting attentions and affections from another human being. Each of us is an absolute individual and has an inherent value which is particularly valuable for the whole of society. Getting to know another human being relies on experiencing someone else’s experience (Nach-liben) and co-experiencing (Mit-liben). In this way, one can immediately become aware of individual ways in which people exist and fulfill themselves (Scheler 1972). This is the presentation of another dimension of cognition, in which attention is paid to self-reflection regarding self-awareness or to what extent a person understands themself. This awareness is a prerequisite for a person that allows another person to understand what he or she thinks, what he or she does not want, and who he or she loves (Scheler 1972). Max Scheler highlighted the value of self-creation of the cognitive and empowering person as an act of creative development and openness towards others. The limitation of human cognition is only love, as a deep feeling that enables one to get to know another person, a process that is always ongoing.

The optimal possibilities of getting to know another person has also been researched by Adam Węgrzecki (1972). According to him, a self-cognitive being is a being who continues to self-define and self-determine and is also cognitively defined by external influences. Self-determination may be the result of another person’s encouragement or instruction (Węgrzecki 1972). This self-knowledge is dependent on recognising other humans and becomes the defining characteristic of human nature that all humans share and mutually acknowledge (Węgrzecki 1972). It is due to contact with other people that the entire process of self-discovery takes place. Openness to others leads to the enrichment of self-development. As noted by Węgrzecki (1972), one’s own being manifests itself in meeting other people at various levels, located between the poles of fullness/incompleteness, adequacy/inadequacy and authenticity/inauthenticity. Coexistence and co-participation have become important and irreplaceable in the cognitive dimension. Through experience we can gain an insight into the specific dimension of human existence and develop understanding of individual uniqueness (Węgrzecki 1972).

### A Change of Approach in Relation to the Cognition of People with Autism Spectrum Disorders

Epistemology is a theory of cognition, a field dealing with cognition and knowledge, introducing knowledge in two ways: rationalist theory and empiricism (Benton and Craib 2003).

Rationalist theory is based on human understanding of the world (the science disciplines derive from this trend) in the strict sense, as a product of investigation – Cartesian Cogito ergo sum – which requires testing and proving to verify the cognition.

The other way uses empiricism, where people acquire knowledge based on their intuitions, common sense and previous experience (Ziemiński 2001; Hetmański 2007; Woleński 2007). This approach, however, reveals the diversity and specificity of the cognitive process. According to theory, individual cognition results in a multitude of possibilities, ways of explaining sensory phenomena that seem to be the same, and this offers the need for further research into individual responses. Humans seek to find regularity and routine in their social interactions (Burrell and Morgan 1979). This is illustrated by a selection of specific traits identified by autism, or by Rett, of specific behaviors occurring in the young females from the studied group of children by Kanner. A different approach is presented by Schütz (2008), who turned his attention to the impossibility of objective cognition. The idea is you need to experience the social phenomenon by participating in it in order to be able to describe it. These opposing approaches indicate the clash between objective and subjective cognition. Presented by Gareth Morgan’s (1980, 1981, 1983, 2005) metaphor, epistemological cognition occurs due to the human’s need to commit to the world. Metaphors are used when a person tries to understand a fragment of the experienced reality with the help of another fragment (G. Morgan 2005). Knowledge is made through metaphors that can act as a multi-element source of cognition at the semantic (language) or psychological (manifestation of experiences) level. Theories and explanations regarding the life of the organisation are based on metaphors that start from seeing and understanding the organisation in a characteristic but partial way. Metaphors are often regarded as a way to embellish discourse, but their meanings are much greater. The use of metaphor is, in fact, the consequence of “thinking” and “way of seeing” that pervade our understanding of the world in general (Morgan 2005). Distortion of understanding can occur, however, when subjectivity of cognition becomes confused with metaphor. Morgan warns that one of the interesting aspects of a metaphor is that it always gives a one-sided view of a given case. Putting some interpretations to the fore, it pushes others aside (Morgan 2005).

A transformational-generative theory approach can be one way to investigate the cognition of a person with ASD (autistic spectrum disorder) and this method, which focuses on metaphor, has been developed by Lakoff. Language and cognition are related through mutual influence. Metaphor, in Lakoff’s view, affects perceptions, thoughts and actions (Krzeszowski 2010) in responding to experiences. St Thomas Aquinas presents spirituality metaphorically in relation to material experience while C. S. Lewis emphasises that reality is found not as an objective/external reality but as it appears in human experience. It is the knowledge and experience that create the metaphors, which result from cognition and thinking; shaping/perception/taking over systematic coherent conceptual systems based on experience; shaping language as a tool of cognition (Krzeszowski 2010). The use of metaphors allow one to present their own experiences and experiences which, according to Lakoff (Lakoff and Johnson 2010: 41), can be of a structural nature (when one concept gives a different metaphorical structure) or an orientational one (showing the spatial orientation but having its location in physical and cultural experience). An important element of describing phenomena is the environment – the culture in which the perception of reality is made. The most basic values in a given culture are coherent with the metaphorical structure of the most basic concepts occurring in this culture (Lakoff and Johnson 2010). Thanks to ontological metaphors, it becomes possible to determine the aspects of a situation or a phenomenon, a generalisation and an abstraction to identify common elements as metaphors for things and substances (e.g. quantification, generalisation of goals, etc.) (Lakoff and Johnson 2010), a metaphor of a container is that it is a term for space; personification (as giving human attributes to physical objects); metonymy (as giving human characteristics to phenomena, such as theories and diseases) (Lakoff and Johnson 2010). This approach seems particularly important in analysing the statements of people with ASD. Metaphors can often be used in conversations with them and are often referred to as a metaphorical message, a metaphorical language.

An interesting approach was presented by Maria Gołębiewska (2017), who pointed to various ways of capturing the metaphor as a figure of language and cognition. Appearing in Jacques Derrida’s texts, a metaphor is:

- A source of the concept (a metaphorical combination of words as the source of the third meaning, then taken in the concept)
- The opposite of the concept (myth and metaphor opposed to science and the concept – metaphor as the truth of myth)
- A combination of words and comparison based on analogy (analogy in a metaphor as the extraction of ‘common sense’ compiled words)
- A comparison giving the possibility of orientation (indication of meaning and focus of attention)
- A source of sense, as a means to giving the possibility of making sense (giving sense to existence as an attempt to adjudicate on existence)
- A comparison, extracting one meaning to hide another (a metaphor opposed to a transparent concept) (Gołębiewska 2017).

Using these insights, there is a way that the often hidden/unrecognised essence of statement can be found and interpreted as an individual and specific construct finding the essence, often hidden/unrecognised sense in the statements, which are an individual and specific construct. By referring to Heidegger (Gołębiewska 2017), one can refer to the truth understood as the unconcealedness of being, as aletheia, that is, what is revealed, while being hidden. According to Heidegger, this truth, extracted in language through metaphor, allows an entity conscious of its own being to capture the meaning of its own existence. Truth understood in this way is meaning extracted through metaphor, it is discovered in language and in the unit itself, and at the same time present due to the creative activity that characterises both language and human existence (Gołębiewska 2017). It is this statement that determines the potential for a metaphor to read human experiences and seek understanding in a confusing world – an integral cognitive system shaping a differently perceived reality (often referred to as Others, Different).

We can use these theories and concepts as a means for accessing the minds of people with ASD, to obtain a deeper understanding of their perceptions, self-reflections and sense of self. Lakoff’s theory allows for analysis of the obtained statements in a non-literal way, instead presented having taken the subject’s cultural context into account, as well as specificity, unconventionality, cognitive processes, perception levels and feelings regarding reproduction. Many matters of self-perception relate to a person’s innate biology, their gender and age, for example. Therefore, analysis of subjects’ statements must take this into account objectively and without preconceived bias. For most people, metaphor is the centre of a poetic imagination and a rhetorical ornament and, therefore, something unusual that does not appear in everyday language. But researchers have found that metaphor is used daily in thoughts and actions. The system of concepts that man usually uses to think and act is essentially metaphorical (Lakoff and Johnson 2010). It should be noted that the theory of these authors is subject not only to criticism, but to continuous evaluation. This has been ongoing since the concept was first proposed in the 1980s (Tokarz 2000). Many aspects of the theory have been criticised, yet there is general agreement regarding religious concepts and emotional states (Kiklewicz 2006). However, as Nowakowska-Kempna (Kiklewicz 2006) observes, apart from idiomatic nominations of feelings in Polish, there are many other means of verbalisation – lexical, syntactic, morphic and even phonetic.

There is further criticism of the application of this theory, as noted by Gemel (2016), especially regarding the conceptualisation of abstract experiences. However, the essence, which he draws attention to, is the possibility, through the use of metaphors, to enrich the perception of the surrounding world, in particular abstract and heavily subjectivised spheres of psychic experience. However, the employment of the metaphor as the only way to shape these spheres, defining the process of their conceptualization, is unlikely (Gemel 2016).

The author agrees with Kiklewicz (2006), who claims that contemporary cognitive theory does not coincide with traditional linguistic theory and is directed towards hypostasia (attempts to reduction a description, focus on one aspect of an object with a simultaneous break from previous methodological and heuristic studies). Above all, it should be noted that metaphorical projection is but one module in a multifaceted, multi-level system of conceptualisation, at the base of which are proposed semantic structures (Kiklewicz 2006).

Another important element is the universality of using metaphors in communication, which makes it one of the basic forms of information transfer. This is important, and not only for everyday communication, and so has become an important foundation of the research.

The transition from empathy (as empathy in the eidetic^2^-phenomenological definition of Husserl's cognition of man), the social influence factor, cognition in relation to one’s own experiences and the cognition of the other being, becomes an important factor when conducting research into personality and character. It also affects the way understanding is viewed. This approach paves the way to a new view of autism/autism spectrum disorders that deviates from assessment, classification or labelling. Naming and evaluating term changes that indicate significant characteristics (autism, early childhood autism, autism spectrum disorder), as well as categorisation in the terminological range (as diseases, disorders, otherness, neurodiversity, etc.) is a typical example of the metaphors most appropriate to the state of knowledge, and the scope of analysing these terms. The current state of knowledge and ongoing social changes allows the author of this paper to test the approach using a new perspective and introducing a new scope of research on the specificity used in the definition of autism and behaviours presented by people diagnosed as suffering from this condition.

A change in the approach to investigating the perceptions of a person with ASD reflects the need to keep pace with the continuously developing understanding of this disorder. Until now, society has defined it based on observation and the creation of terrestic models such as early childhood autism/autism. The mere change of the name to spectrum disorders, as well as the introduction of degrees – depth of specific behavior occurring (in DSM 5, ICD 11), indicates the need to move away from the original definition in favour of looking for new perspectives and inclusion prospects (see Błeszyński 2018; Błeszyński 2019).

#### Understanding – Perception of Autism/autism Spectrum Disorder

During the research process, the author came across classification definitions of autism that relate to the variation associated with the behaviour that occurs. The main elements of the description are behaviours that diverge from specific indicators – concerning, above all, social behaviors, including communication (communication and social interactions) and activities undertaken (and related limitations, repeated patterns of behavior or activities).

The resulting concepts have been based on a clinical approach, which is intended primarily to sublimate the characteristic symptoms that enable Autistic people to be distinguished, to determine the severity and possible consequences of occurrence. This approach applies to both functional and clinical analysis, and the purpose is to clarify the deviation from the norm. The essence of the term “norm” is defined in dictionaries. The Dictionary of the Polish language defines the term as “*a quantity, a measure, a limit prescribed as required or binding in some respect*” (Polish Dictionary 2019). The question is whether it is necessary to set widely understood norms today. Tomasz Leś claims that pedagogy is impossible as a science without norms. But it is worth adding that just as it is difficult to justify the fact that pedagogy should not contain normative sentences, so it is difficult to indicate a specific normative system on which it should be based on (Leś 2011: 56). The norm in pedagogy, and especially in special pedagogy, is used not as a boundary but as a reference point. Such an approach can be observed in relation to any deviations, both to human development as well as disharmony and existing deficits.

A different approach is the inclusive, humanistic trend, which allows to reverse the definition of a norm defined by those described. Currently, especially in the personalistic approach, the problem of social awareness, as well as self-awareness of individual members of a given society is raised, in particular those rejected, excluded and marginalised so far, which often led to deprivation of their rights and, consequently, to the idea of euthanasia. Thanks to social changes, as well as the possibilities of the mentioned social inclusion, people with deficits (e.g. people with cerebral palsy, high-functioning autism, etc.) hitherto facing exclusion, found themselves in the circle of social inclusion. The possibility of establishing contact with these people, as well as their effectiveness in the social structure, allows us to look at their hitherto unilaterally formulated statements and definitions regarding the phenomenon of autism.

## Methods

The responses to the question what autism is in the netnographic interview brought very important information requiring differentiation due to the age of the respondent (division into four age groups), as well as gender (declared by the respondents). The research was conducted on 168 subjects (94 women and 72 men). All of the subjects agreed to participate in the Internet interview, addressed to closed groups of autistic individuals. The interview comprised twenty-one questions: seven multiple-choice questions and fourteen open-ended queries (What is Autism?). In the process of analyses, narratives were first grouped qualitatively (inductively), and then analysed by hierarchical grouping (Massart et al. 1997) quantitatively, with division into groups in order to interpret the results qualitatively. However, the answers themselves form important statements that can be analysed. As part of the analysis of the collected material the statements were grouped. This provided not only for examining the terms and phrases used by the respondents but also for identifying their intensity and respective contexts. Analysing source literature that has been published so far, the author has mainly found research papers analysing ASD individuals. Natali Walter’s work presents the results of the research on the image of children with ASD based on the published blogs with opinions and memories of their parents (Walter 2016). Other netnographic research conducted by Sandra Jane Ellis (2014) focused on teachers and ASD students and referred only to education. Sarah Parsloe’s (2015) paper, based on 561 posts and ten netnographic interviews, focused on identity of ASD individuals. The author has been unable to find any netnographic research on the perception of ASD; however, related studies that are available can significantly contribute to obtaining the actual information from people with ASD. The primary reason for using the netnographic method was the opportunity it creates in reaching this part of the population more effectively. People with ASD have difficulty in establishing and maintaining communication, and organising the obtained information. They also have certain limitations associated with the face-to-face contact. By enabling a manner of communication that is more acceptable to them, the Internet allows us to gain access and collect information through closed online forums dedicated to people with ASD.

Consequently, the respondents in this study were highly functioning people with ASD, associated in online forums dedicated exclusively to people with ASD. These are often homogeneous self-help groups where people without the ASD diagnosis are not admitted (Silberman 2015, p. 443).

All procedures performed in studies involving human participants were in accordance with the ethical standards of the institutional and/or the national research committee and in accordance with the 1964 Helsinki declaration and its later amendments or comparable ethical standards (Table 1).

**Table 1:** Results of netnographic interviews with females in relation to age – marked responses in numbers [N = 94]. Source: own research

10–20	Σ	20–29	Σ	30–39	Σ	40–48	Σ	*The feeling of being different, a negative reception*
Me/ part of me	6	Being different	10	Being different	10	Developmental dis order	5	
Different behavior	4	Disease, disfunction	7	Me	6	Being different	4	
Being different	2	Lack of acceptance	4	Disease	4	Different type of social development	4	
Sensitivity	2	I don’t know	3	Hard to say	3	Me, part of me	3	*Connected with identity, social as well*
Disorder	1	Way of development	2	sensitivity	3	I don’t know	1	
Feature	1	Acceptance, friends	2	Limitation, difficulty	2			
Isolation	1	My own word	1	Way of understanding	1			
		Social disorder	1					
*Different sensitivity of self-awareness*		*As a disorder not accepted by the environment*		*Being different as a result of cognition and ability to feel differently*		*Different type of self-development impairing social functions*		

## Results

**Fig. 1:**
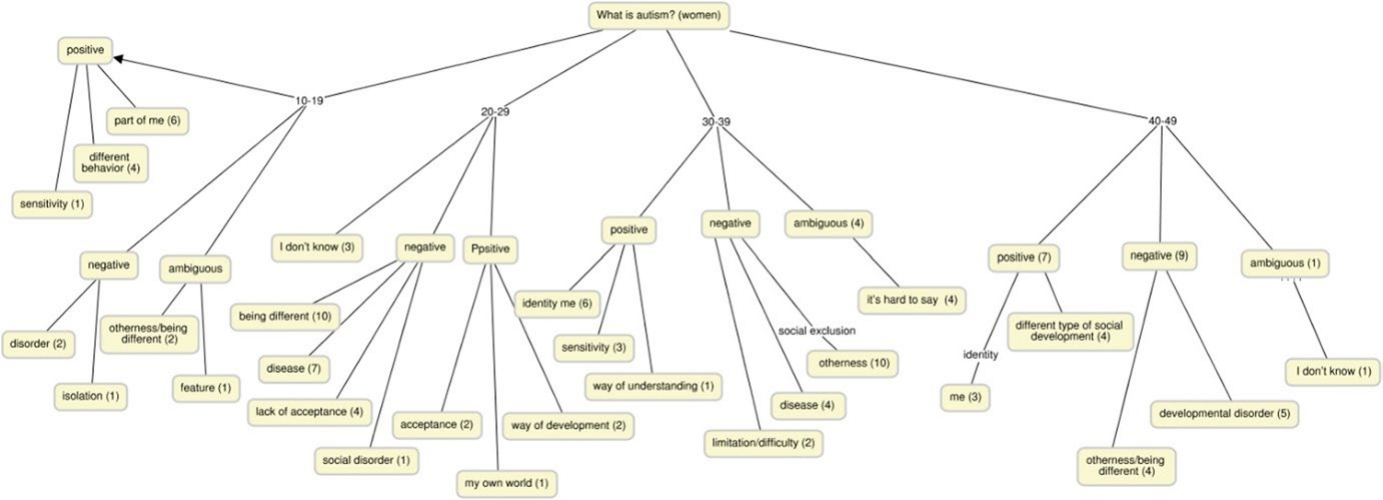
Results of netnographic interviews with females in relation to age – graphic generalisation [N = 94]. Source: own research

The chart below shows a wide age range amongst the respondents, regardless of the number of people participating in the interview. Even taking individual subjectivity into account, it was still possible to divide into positive, negative and ambiguous terms in all age ranges (Fig. 1).

**Fig. 2:**
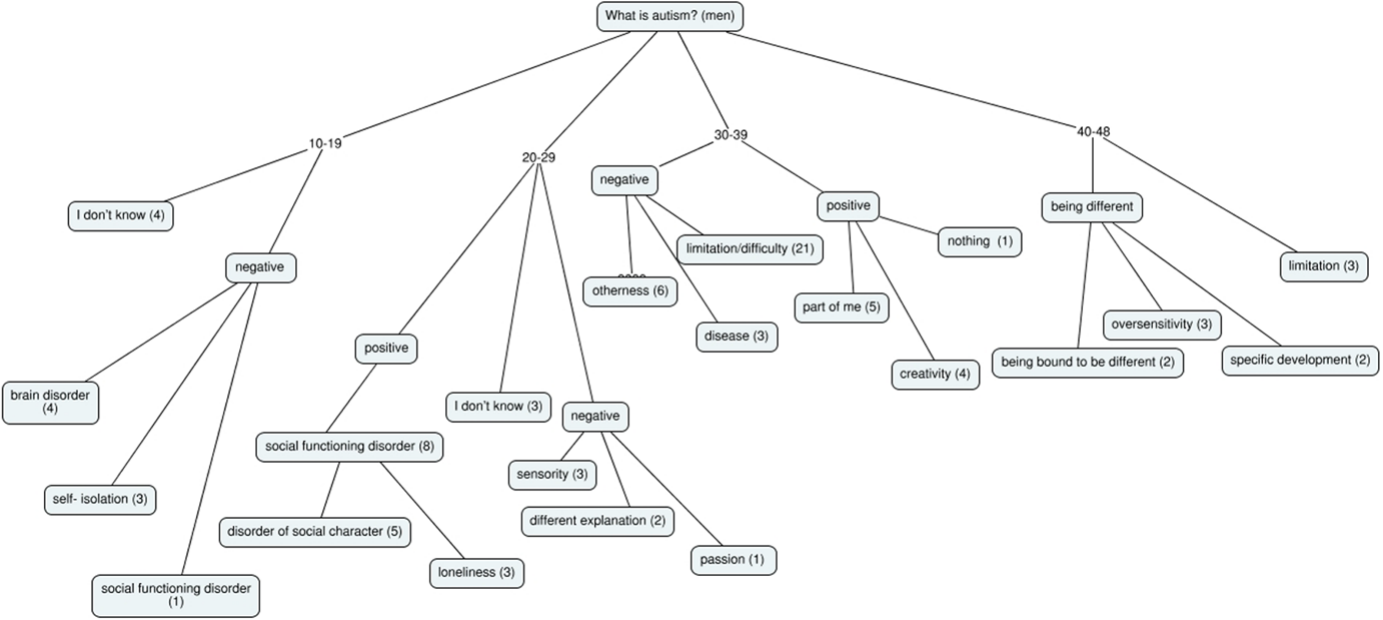
Results of netnographic interviews with males in relation to age – graphic generalization [N = 72]. Source: own research

The most frequent responses are personal, and the most common are:

- **Negative**: disorder, illness, limitation, difficulty, isolation, lack of acceptance
- **Positive**: part of me (identity), sensitivity, different behavior, acceptance, way of understanding, difference
- **No clear statement**: I do not know, it’s hard to say, difference, feature (Table 2, Fig. 2)

**Table 2:** Results of netnographic interviews with males in relation to age – marked responses in numbers [N = 72]. Source: own research

10–20	Σ	20–29	Σ	30–39	Σ	40–48	Σ	*Feeling different with a limited perception of social functions *
Brain disorder	4	Social character	5	Difficulty	21	Limitation	3	
I don’t know	4	Sensoriality	3	Being different	6	The specificity of development	2	
Self-isolation	3	Loneliness	3	Part of me, identity	5	Conviction for dissimilarity	2	
Disease	2	Disease	2	Creativity	4	Oversensitivity	1	*Disease associated with different perception—not always worse*
Social incompleteness	1	Different explanation	2	Disease	3	Something familiar	1	
		I don’t know	1	Nothing	1			
		Passion	1					
*Disorder difficult to determine due to isolation*		*Difference causing isolation*		*Difficulty resulting from one’s own different perception*		*Limitation characterized by different cognition*

Undoubtedly, it is important to take into consideration the social context that the person with this condition is most frequently exposed to. Especially in relation to children, stigmatising and discriminating terms such as “illness” and “disorder” are used. The statements presented by the respondents are an indication of their perception of how society sees them, through the prism of stigmas, negative distinctions and a result of an institutionalised language, most often appearing in their environment.

In the case of men, there are definitions reflecting negative features in all age groups. Most answers regarding autism are personal:

- **Negative**: a disorder that primarily affects social functions, otherness.
- **Positive**: connected with oneself (identity), sensory, other explanation, passion.
- **No clear statement**: I do not know, specificity of development.

The results presented in the numerical comparison, as well as the generalisation of the results obtained, are presented below.

When compiling the data, it can be presented in the form of numerical data and the amount of data broken down into the information obtained in the interview with regard to age, and then generalised.

The age of respondents is an important element in their views about autism. Above all, there is a negative connotation of the word itself and related behaviours that increase with age. Presenting the features described as a personal experience for a social approach, ranging from partial sensitivity, through social reception to diversity related to individual development, is a characteristic trait.

In the case of men, negative statements are primarily pertaining to how respondents view their own characters, focusing mainly on social interactions but also on their perception of incoming stimuli. With age, we can observe the evaluation of their views of autism from being socially isolated towards a personal experience and building one’s own identity. With age, the perception of autism matures into engagement in interaction and development of adaptation whilst maintaining their unique cognition. It can be observed that ASD individuals are able to become socially mature but still hold negative views of their condition – autism is a dissimilarity inherent in them and so does not allow them the full development of social participation.

Regardless of maturity, negative perceptions of autism, such as dissimilarity, otherness and traits that prevent fuller social consolidation prevail. Attention is drawn to the fact that these characteristics are perceived as personal features, associated with identity, influencing the way they are seen by society.

By analysing the intensity of choices, it can be said that regardless of the age of the respondents, the impairment of social functioning is the most significant problem (mainly the reception). Additionally, autism is presented as dissimilarity, however, importantly, it is not always associated with the ameliorative (only) reception.

## Conclusions

Analysis of the generalised results from the interviews of the gender groups offers a way of accessing a different perception of autism. Starting from reception with respect to social experience, through the development of the reception in relation to the environment, followed by forming the basis of their own experiences. In the oldest age group, respondents show their own individual approach to autism. Such a process can be defined as maturing to one’s own understanding on the social ground. This transition can be seen as an attempt to participate more fully in social life but with a self-conscious image. It is justifiable here to refer to the experiences presented by Liane Willey (2018), who called this attitude a pretence of normal – that is, adjusting and counteracting the distinction from the rest of society (Table 3).

**Table 3:** Results of netnographic interviews with females in relation to age – descriptive generalisation: Source: own research

10–20	20–29	30–39	40–48	generalization of the results
*A disorder difficult to determine connected with isolation*	*Difference causing isolation*	*Difficulty resulting from one's own different perception*	*Limitation characterized by different cognition*	*Being different with negative connotations. A different, limited perception of social functions*
*Identitatively different sensitivity*	*As a disorder not accepted by the environment*	*Being different as a result of cognition and ability to feel differently*	*Different type of self-development impairing social functions*	*Disease associated with a different perception – not always worse. Perception connected with being different, also socially*
*Environment and cognition*	*Environment*	*Cognition*	*Cognition and environment*	

The presented analyses indicate an important aspect of the self-awareness of people with ASDs regarding dissimilarities from the rest of society as well as attempts to limit this distinction. As an environment society tends, most often with the help of institutions, to determine utilitarian behaviours (in extreme cases of subordination) as well as re-evaluate, giving excessive importance to individualism (often in extreme terms) – which, in the case of people with a diagnosis of autism, can lead them to form a sense of uniqueness against the social background. In the case of the presented research, such extreme approaches are also difficult as they do not facilitate the formation of a mature identity in society. The results grouped according to age and gender allow for noting the differences stemming from social adaptation, which seems to be more advanced in the case of women, who have fewer difficulties with communication and are more open to social interaction.^3^

Referring to research conducted on people with ASD regarding self-evaluation, it is worth paying attention to broadening awareness of the spectrum itself. The research conducted by Schriber et al. (2014), using a cohort of 37 adults with ASDs and typically developing persons (N = 50), confirmed that people from the spectrum have insight into themselves (sense of identity) and are able to initiate their social experience (similar results showed in both groups). People with autism spectrum are aware and recognise states of behavior of other people and can define them as traits. On this basis, the validity of this research can be confirmed. Rejecting the inability to conduct such research, as the author points out, it can be concluded that people with autism have different personality traits and a reasonable degree of insight into their relative position, with a tendency to systematically abuse their assessments. A better understanding of personality and self-assessment processes in people with ASD can have important implications for the understanding and treatment of autism, as well as for a better understanding of the interface between personality, self-insight and overall pathological functioning (Schriber et al. 2014).

## Summary

The results of the study drew attention to a few important aspects. The legitimacy of testing using netnographic intelligence, using multimedia resources, was confirmed by Kitson-Reynolds et al. (2015), pointing to the legitimacy of using technical means in communication and learning – not requiring direct contact (e.g. face to face). Despite difficulties connected with qualitative analysis (especially objectivism of the collected material) of ASD individuals, this type of analysis seems to be the most effective tool. Another important aspect of the study was to involve ASD individuals in presenting their views in the study and to prevent their alienation (activity in closed Internet forums). The material collected in the course of this research was acquired directly from autistic individuals, not from specialists in the field (psychologists, doctors, pedagogues, sociologists). It is a transition from outer knowledge (from specialist) to inner perception (by autistic people) organised methodically by the researcher (research optimalisation). It can be treated as a new quality or new in-depth knowledge acquired directly from the interested subjects.

The conducted research allowed the author to observe that autism is more of a function of exaggerated features/traits which are common in so-called neurotypical individuals. This affects social functioning, especially communicative competences of speech and language.

## Footnotes

1 The material presented in the article is a part of a broader research prepared for a publication of the NCU in Torun.

2 Encyklopedia eidetics [gr. eidētikós ‘ideal’, ‘formal’ < eídos ‘image’, ‘person’, ‘pattern’], in phenomenology, knowledge of what is important. The term was introduced by E. Husserl: in his view phenomenology is eidetiv, i.e. it searches for stable/unchanged features Ina constantly changing content, it focuses on the core problem; Husserl used a triangle to explain his view as definition of this figure is independent of the size of sides of this triangle. (https://encyklopedia.pwn.pl…; 7.09.18).

3 More about these considerations in a broader publication of NCU in Torun in 2020.
